# Cardiac output drop reflects circulatory attrition after Fontan completion: serial cardiac magnetic resonance study

**DOI:** 10.1093/ehjimp/qyad039

**Published:** 2023-11-27

**Authors:** Sara C Arrigoni, Rolf M F Berger, Tjark Ebels, Douwe Postmus, Elke S Hoendermis, Paul H Schoof, Tineke P Willems, Joost P van Melle

**Affiliations:** Department of Cardiothoracic Surgery, University of Groningen, University Medical Center Groningen, Hanzeplein 1, PO Box 30.001, Groningen 9700 RB, The Netherlands; Department of Pediatric Cardiology, University of Groningen, University Medical Center Groningen, Groningen, The Netherlands; Department of Cardiothoracic Surgery, University of Groningen, University Medical Center Groningen, Hanzeplein 1, PO Box 30.001, Groningen 9700 RB, The Netherlands; Department of Epidemiology, University of Groningen, University Medical Center Groningen, Groningen, The Netherlands; Department of Cardiology, University of Groningen, University Medical Center Groningen, Groningen, The Netherlands; Department of Pediatric Cardiac Surgery, University of Utrecht, University Medical Center Utrecht, Utrecht, The Netherlands; Department of Radiology, University of Groningen, University Medical Center Groningen, Groningen, The Netherlands; Department of Cardiology, University of Groningen, University Medical Center Groningen, Groningen, The Netherlands

**Keywords:** cardiac magnetic resonance, Fontan circulation, univentricular physiology, congenital heart disease

## Abstract

**Aims:**

Cardiac magnetic resonance (CMR) imaging is a main diagnostic tool in the follow-up of Fontan patients. However, the value of serial CMR for the evaluation of Fontan attrition is unknown. The aim of this prospective study of serial CMR is to describe the analysis of time-dependent evolution of blood flow distribution, ventricular volumes, and function in patients after Fontan completion.

**Methods and results:**

In this prospective single-centre study, between 2012 and 2022, 281 CMR examinations were performed in 88 Fontan patients with distribution of blood flows, measurements of ventricular volumes, and ejection fraction. Linear mixed model regression for repeated measurements was used to analyse changes of measurements across serial CMR examinations. During a time interval of 10 years, the median number of CMR per patient was 3 (range 1–5). Indexed flow of ascending aorta, caval veins, and pulmonary arteries decreased significantly across serial CMR examinations. Although a decrease of mean indexed aortic flow (3.03 ± 0.10 L/min/m^2^ at first CMR vs. 2.36 ± 0.14 L/min/m^2^ at fourth CMR, *P* < 0.001) was observed, ejection fraction did not decline (50 ± 1% at first CMR vs. 54 ± 2% at fourth CMR, *P* = 0.070). Indexed ventricular volumes did not differ significantly across serial CMR examinations.

**Conclusion:**

The decrease of indexed aortic and cavopulmonary flows reflects the attrition of univentricular circulation and can be detected by means of serial CMR. Ventricular systolic dysfunction does not contribute significantly to this attrition. In order to detect significant change of indexed aortic flow, we recommend performing serial CMR as routine practice in the Fontan population.

## Graphical Abstract

**Graphical Abstract:**
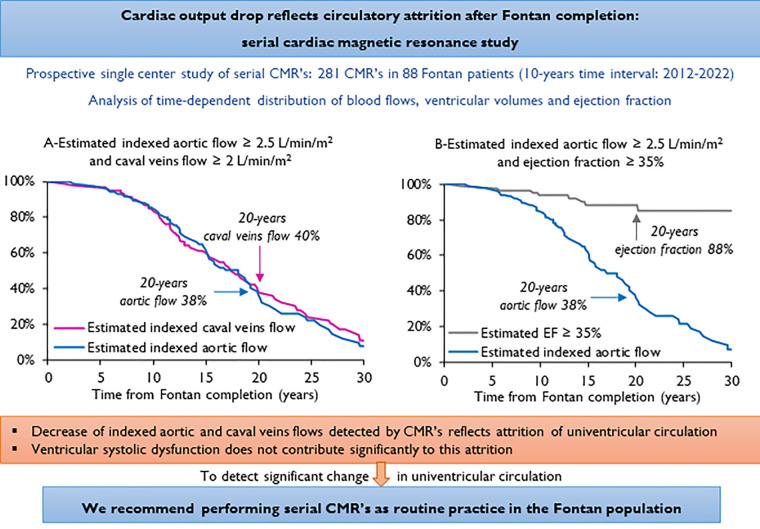
Decrease of indexed aortic and caval veins flows (Kaplan–Meier curves, Graph A) detected by means of serial CMR reflects attrition of univentricular circulation. The contribution of ventricular systolic dysfunction to this attrition is limited: Kaplan–Meier curves of indexed aortic flow and EF diverge remarkably (Graph B). We recommend performing serial CMR as routine practice in Fontan population to detect significant haemodynamic change. CMR, cardiac magnetic resonance; EF, ejection fraction.

## Introduction

Patients with almost any functionally single cardiac ventricle are currently palliated by a Fontan circulation. The hallmark of a Fontan circulation is the redirection of systemic venous blood directly to the pulmonary arteries, separating it from the pulmonary venous return. Since its introduction, several modifications have been made. In 1988, de Leval *et al*. introduced the total cavopulmonary connection as an alternative to the atriopulmonary connection originally described by Fontan and Kreutzer.^1,2^ Despite improvements in outcome,^3^ the evolution of the Fontan circulation and the several late complications (cyanosis, arrhythmia, Fontan-associated liver disease, protein-losing enteropathy, and plastic bronchitis) are worrisome.^4^ Several factors may contribute to late Fontan failure, like remodelling of the pulmonary vascular bed, obstruction within the Fontan circuit, atrioventricular valvar regurgitation, and systemic ventricular failure. Cardiac magnetic resonance (CMR) imaging allows for a comprehensive assessment of the Fontan physiology by means of measuring ventricular volumes and function, blood flow, and quantification of valve regurgitation.^5,6^ In the consensus paper of the imaging working group of the Association for European Paediatric and Congenital Cardiology and the Cardiovascular Magnetic Resonance Section of the European Association of Cardiovascular Imaging, CMR is recommended after Fontan completion for serial follow-up of ventricular function and anatomical assessment of the Fontan pathway.^7^ Therefore, the 2020 guidelines of the European Society of Cardiology stated that CMR should be regularly performed, however, without a rationale for time intervals.^8^ Since 2012, in our department, patients with a Fontan completion undergo standardized CMR at intervals of 2–3 years, as part of their regular follow-up. It is our objective in this single-centre prospective study to describe the blood flow distribution, the ventricular volumes and function, and their time-dependent evolution based on serial CMR. Because indexed ascending aortic flow is equivalent to the cardiac index, the main purpose of this study was to analyse the evolution of indexed ascending aortic flow and its potential determinants.

## Material and methods

### Study population

In this prospective single-centre study, Fontan patients since 2012, from the age of 7 years old, underwent CMR with standardized flow measurements as part of regular follow-up at intervals of 2–3 years. Patients with Fontan circulation who underwent at least one CMR during follow-up were selected from our institutional database, and data were retrospectively collected. Patients with a previous Kawashima operation or who could not undergo CMR because of a pacemaker or defibrillator or had claustrophobia were also excluded. Our Institutional Review Board waived the requirement for informed consent because data were retrospectively collected as part of routine medical care and patients were individually unidentifiable (Medical Ethical Committee of the University Medical Center Groningen number 2022/043).

### Objectives

The aim of this study was to analyse the time-dependent evolution and distribution of blood flows in patients after Fontan completion. Therefore, the following indexed blood flows and volumes were measured, and ratios were calculated:

- Ascending aortic flow
- Superior (SCV) and inferior (ICV) caval vein flows
- Caval veins flow = SCV + ICV
- SCV flow ratio = SCV/(SCV + ICV)
- Right (RPA) and left (LPA) pulmonary artery flows
- Pulmonary artery flow = RPA + LPA
- RPA flow ratio = RPA/(RPA + LPA)
- Collateral flow = aortic—(SCV + ICV)
- End-systolic (ESV) and end-diastolic volumes (EDV)
- Stroke volume (SV)

Furthermore, an analysis of time-dependent evolution of ejection fraction (EF) and heart rate (HR) was performed. All parameters were indexed by means of body surface area using the Haycock formula.

### Cardiac magnetic resonance

**Figure 1:**
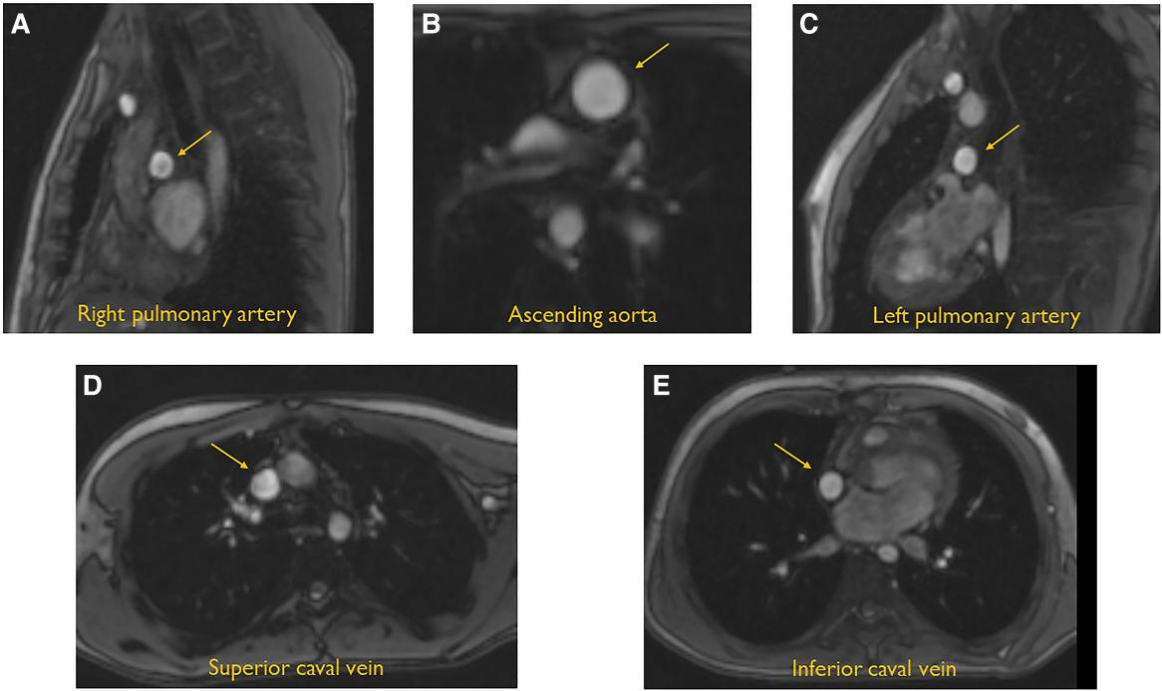
CMR pictures that show the anatomical level of the measured blood flows. (*A*) RPA, direct distal from the cavopulmonary anastomosis and before the first pulmonary artery branch. (*B*) Ascending aorta, 1.5 cm above the aortic valve. (*C*) LPA, between the cavopulmonary anastomosis and the first pulmonary artery branch. (*D*) SCV, below the entrance of the azygos vein, if present. (*E*) ICV, between hepatic vein confluence and right atrium or conduit.

All CMR studies were performed without sedation and without using contrast, on a 1.5 T system (Siemens, Magnetom Avanto, Erlangen, Germany). The CMR protocol for functional analysis included a stack of short-axis slices from the base of the heart to the apex of the heart using cine steady-state free precession with end-expiratory breath holding.^9^ The following scan parameters were used: slice thickness, 6 mm; slice gap, 4 mm; time repetition, 2.7–3.4 ms; time echo, 1.1–1.7 ms; flip angle, 80–90; matrix, 171–192; and voxel size, 1.25 × 1.25 × 8.0 mm and 1.7 × 1.7 × 6.0 mm. Imaging analysis was performed by using commercially available software (Qmass, version 7.6.14.0, Medis Medical Imaging Leiden, the Netherlands). End-systolic and end-diastolic phases were visually selected, using the largest and smallest systemic ventricular cavities on the longitudinal and short-axis views. The contours of the systemic and hypoplastic ventricle, if present, were manually drawn on epicardial and endocardial borders from the most apical to the most basal short-axis slice. The end-systolic and end-diastolic blood volumes were calculated from the endocardial contours; both the volumes of the systemic and hypoplastic ventricles were included except for patients with pulmonary atresia and intact ventricular septum.^10^ EF and SV were calculated from EDV and ESV. HR by measurement of aortic flow was registered. Indexed ascending aortic flow was considered equivalent to cardiac index. Phase-contrast imaging was performed of ascending aorta, both pulmonary arteries, and both caval veins. *Figure 1* shows a composite sample of the anatomical level of each CMR measurement. Blood flow was measured in the ascending aorta, 1.5 cm above the aortic valve (*Figure 1B*) (after a Damus–Kaye–Stansel procedure, above the distal anastomosis), SCV (*Figure 1D*) (below the entrance of the azygos vein if present), ICV (*Figure 1E*) (between hepatic vein confluence and right atrium or conduit, downstream of the level of fenestration, if present), RPA (*Figure 1A*) (direct distal from the cavopulmonary anastomosis and before the first branch), and LPA (*Figure 1C*) (between the cavopulmonary shunt and the first LPA branch).

All flow images were analysed using QFlow (v5.6, Medis Medical Imaging, Leiden, the Netherlands). Automatic background offset correction was performed.

### Statistical analysis

Statistical analysis was performed using IBM SPSS Statistics 28 (IBM Corporation, New York, USA).

Values are reported as mean ± standard deviation. Linear mixed model (LMM) regression for repeated measurements was used to analyse the evolution of blood flow measurements, ventricular volumes, EF, and HR by modelling the regression within the repeated measures over time. Therefore, patients with at least two CMR examinations during follow-up were included in this analysis (76/88 patients). Because only 18/88 patients had five CMR examinations, LMM was performed on a maximum of four serial CMR examinations. In all time-dependent variables, time zero corresponded to the date of the Fontan completion (T0). Repeated measurements were ranked in four temporal classes: first, CMR after Fontan completion corresponded to Time 1 (T1), and so on. The LMM regression was repeated by the paediatric subgroup: 27 patients with age less than 18 years old. To minimize potential confounding by a factor related to the interval range of time between the date of Fontan completion and the first CMR, the LMM regression was repeated by a selected subgroup of 44 patients with the first CMR within 10 years after Fontan completion.

Repeated measurements were analysed either as fixed or as random effects. Pairwise comparisons were based on estimated marginal means. The mean difference ± standard error and 95% confidence intervals (CIs) were reported. A two-tailed *P*-value less than 0.05 was used for statistical significance.

The survival curve of the entire cohort of patients (*n* = 88) for all indexed flows, volumes, and HR with associated point-wise linear 95% CIs was estimated using the Kaplan–Meier estimator. A normal range for cardiac index (≥2.5 L/min/m^2^) was used as a reference limit for the Kaplan–Meier curve of indexed aortic flow.^11^ Because the average collateral flow measured in our study was 0.5 L/min/m^2^, a normal range of indexed caval veins flow ≥ 2 L/min/m^2^ was considered as a reference limit. For the indexed volumes, EF, and HR, the following reference limits were used for the Kaplan–Meier estimator: ESV ≥ 30 mL/m^2^, EDV ≥ 65 mL/m^2^, SV ≥ 35 mL/m^2^, EF ≥ 35%, and HR ≥ 60 bpm.^12^

## Results

### Study population

Our study population comprised 88 Fontan patients and 48/88 (55%) were male. Baseline characteristics are shown in *Table 1*. Tricuspid atresia was the most frequent diagnosis (36%). A morphologically left ventricle was most frequent (81%). Mean age at Fontan completion was 5.6 ± 3.1 years. The right auricle tunnel technique, as previously described by us,^13^ was performed in 21/38 lateral tunnels. In all except two patients, a Gore-Tex® vascular prosthesis was used as extra-cardiac conduit (diameter 16–24 mm). In 45 patients (51%), the tunnel was fenestrated, of which 56% were closed at follow-up.

**Table 1:** Baseline patient characteristics

Baseline characteristics		*n* patients (%)
Gender	Male	48 (55%)
Dominant cardiac chamber morphology	Left	71 (81%)
Cardiac anatomy	Tricuspid atresia	32 (36%)
	Double inlet left ventricle	19 (22%)
	Pulmonary atresia	11 (13%)
	Hypoplastic left heart syndrome	9 (10%)
	Unbalanced atrioventricular septal defect	5 (6%)
	Others	12 (13%)
Age at Fontan completion (years)	<5	54 (62%)
	≥5 and <10	25 (28%)
	≥10	9 (10%)
Fontan completion surgical technique	Atriopulmonary connection	7 (8%)
	Extra-cardiac conduit fenestrated	19 (22%)
	Extra-cardiac conduit not-fenestrated	23 (26%)
	Lateral tunnel fenestrated	26 (29%)
	Lateral tunnel not fenestrated	12 (14%)
	Others	1 (1%)
Decades of Fontan completion	1979–1989	8 (9%)
	1990–1999	23 (26%)
	2000–2009	39 (44%)
	2010–2020	18 (21%)

### Number and timing of CMR

During a period of 10 years (2012–2022), 281 CMR examinations were performed. The median number of CMR per patient was 3 (range 1–5). The mean interval between two consecutive CMR examinations was 2.6 ± 0.1 years. The mean interval between T1 and T3 was 5.0 ± 1.5 years, between T2 and T4 5.1 ± 1.5 years, and between T1 and T4 7.2 ± 1.5 years. The median interval time between T0 and T1 was 9.3 ± 7.6 years (range 1.6–33.6 years).

### Cross-sectional analyses (T1–T4)

*Tables 2* and *3* show respectively all measured mean indexed flows, ratios, mean EF, HR, and indexed flows at four temporal classes.

**Table 2:** Mean indexed flows (L/min/m^2^) and flow ratios at temporal classes

Temporal classes	Mean at T1 (CI 95%)	Mean at T2 (CI 95%)	Mean at T3 (CI 95%)	Mean at T4 (CI 95%)
Number of CMR median years from Fontan completion	76 9.34 ± 7.9	76 11.16 ± 7.68	58 15.18 ± 7.48	41 18.04 ± 7.87
Aortic flow	3.03 ± 0.10 (2.83–3.24)	2.80 ± 0.11 (2.59–3.01)	2.66 ± 0.12 (2.42–2.89)	2.36 ± 0.14 (2.08–2.63)
LPA flow	1.18 ± 0.05 (1.07–1.28)	1.11 ± 0.05 (1.01–1.21)	0.92 ± 0.06 (0.80–1.05)	0.77 ± 0.07 (0.63–0.91)
RPA flow	1.30 ± 0.07 (1.16–1.44)	1.31 ± 0.07 (1.16–1.44)	0.96 ± 0.08 (0.79–1.12)	0.81 ± 0.10 (0.61–1.00)
Pulmonary arteries flow	2.58 ± 0.14 (2.31–2.85)	2.63 ± 0.14 (2.35–2.90)	1.90 ± 0.16 (1.59–2.22)	1.57 ± 0.19 (1.20–1.94)
RPA ratio	0.52 ± 0.01 (0.49–0.55)	0.54 ± 0.01 (0.51–0.57)	0.51 ± 0.02 (0.47–0.54)	0.51 ± 0.02 (0.47–0.55)
SCV flow	1.01 ± 0.05 (0.90–1.11)	0.87 ± 0.05 (0.76–0.97)	0.72 ± 0.06 (0.60–0.83)	0.61 ± 0.07 (0.47–0.75)
ICV flow	1.64 ± 0.07 (1.50–1.79)	1.53 ± 0.07 (1.38–1.68)	1.28 ± 0.08 (1.12–1.44)	1.20 ± 0.10 (1.01–1.40)
Caval veins flow	2.66 ± 0.11 (2.45–2.87)	2.39 ± 0.11 (2.18–2.60)	1.99 ± 0.12 (1.76–2.23)	1.81 ± 0.14 (1.53–2.09)
SCV ratio	0.39 ± 0.02 (0.36–0.42)	0.37 ± 0.02 (0.34–0.40)	0.35 ± 0.02 (0.32–0.38)	0.33 ± 0.02 (0.29–0.38)
Collateral flow	0.40 ± 0.09 (0.23–0.57)	0.42 ± 0.09 (0.24–0.60)	0.66 ± 0.10 (0.47–0.85)	0.53 ± 0.12 (0.31–0.76)

CI, confidence interval; ICV, inferior caval vein; LPA, left pulmonary artery; RPA, right pulmonary artery; SCV, superior caval vein; T1, T2, T3, and T4, time of 1st, 2nd, 3rd, and 4th CMR.

**Table 3:** Mean ejection fraction, heart rate, indexed end-diastolic and end-systolic volumes, and indexed stroke volume at temporal classes

Temporal classes	Mean at T1 (CI 95%)	Mean at T2 (CI 95%)	Mean at T3 (CI 95%)	Mean at T4 (CI 95%)
Number of CMR median years from Fontan completion	76 9.3 ± 7.9	76 11.2 ± 7.7	58 15.2 ± 7.5	41 18.0 ± 7.9
EF (%)	50 ± 1 (47–52)	51 ± 1 (49–54)	52 ± 1 (49–55)	54 ± 2 (50–57)
Heart rate (bpm)	76 ± 2 (73–79)	74 ± 2 (71–77)	73 ± 2 (69–76)	69 ± 2 (65–73)
Index EDV (mL/m^2^)	93.6 ± 3.9 (86.0–101.1)	89.7 ± 3.9 (82.1–97.3)	90.3 ± 4.4 (81.5–99.0)	84.3 ± 5.2 (74.0–94.7)
Index ESV (mL/m^2^)	48.9 ± 3.0 (43.1–54.8)	46.0 ± 3.0 (40.2–51.8)	46.0 ± 3.4 (39.2–52.7)	40.7 ± 4.0 (32.8–48.7)
Index SV (mL/m^2^)	44.3 ± 1.6 (41.3–47.4)	44.6 ± 1.6 (41.5–47.7)	45.4 ± 1.8 (41.9–49.0)	44.3 ± 2.1 (40.1–48.5)

bpm, beats per minute; CI, confidence interval; EDV, end-diastolic volume; EF, ejection fraction; ESV, end-systolic volume; SV, stroke volume; T1, T2, T3, and T4, time of 1st, 2nd, 3rd, and 4th CMR.

### Evolution over time: LMM

*Table 4* shows the LMM regression of indexed flows and ratios based on the four temporal classes. A significant decrease in all indexed flows was observed between T1 vs. T3, T2 vs. T4, and T1 vs. T4. Also, a significant decrease in SCV flow ratio was found comparing T1 vs. T4 (mean difference 0.05 ± 0.03, *P* = 0.045). No significant difference was found in the RPA flow ratio across the temporal classes.

**Table 4:** Linear mixed model regression for indexed flows and flow ratios

	Mean difference indexed flows (L/min/m^2^) and flow ratios between temporal classes											
	T1 vs. T2 (CI 95%)	*P*-value	T2 vs. T3 (CI 95%)	*P*-value	T3 vs. T4 (CI 95%)	*P*-value	T1 vs. T3 (CI 95%)	*P*-value	T2 vs. T4 (CI 95%)	*P*-value	T1 vs. T4 (CI 95%)	*P*-value
Aortic flow	0.24 ± 0.15 (−0.06–0.53)	0.113	0.14 ± 0.16 (−0.17–0.46)	0.372	0.30 ± 0.18 (−0.07 −0.66)	0.108	0.38 ± 0.16 (0.07–0.69)	**0**.**017**	0.44 ± 0.18 (0.09–0.79)	**0**.**013**	0.67 ± 0.17 (0.33–1.02)	**<0**.**001**
LPA flow	0,.07 ± 0.08 (−0.08–0.22)	0.366	0.18 ± 0.08 (0.02–0.34)	**0**.**024**	0.16 ± 0.09 (−0.03–0.34)	0.098	0.25 ± 0.08 (0.09–0.41)	**0**.**002**	0.34 ± 0.09 (0.17–0.52)	**<0**.**001**	0.41 ± 0.09 (0.23–0.59)	**<0**.**001**
RPA flow	−0.01 ± 0.10 (−0.22–0.19)	0.894	0.36 ± 0.11 (−0.14–0.58)	**0**.**001**	0.15 ± 0.13 (−0.11–0.40)	0.253	0.34 ± 0.11 (0.13–0.56)	**0**.**002**	0.51 ± 0.12 (0.26–0.75)	**<0**.**001**	0.49 ± 0.12 (0.25–0.74)	**<0**.**001**
Pulm. arteries flow	−0.05 ± 0.20 (−0.43–0.34)	0.817	0.72 ± 0.21 (0.30–1.14)	**<0**.**001**	0.33 ± 0.25 (−0.15–0.82)	0.178	0.68 ± 0.21 (0.26–1.09)	**0**.**002**	1.06 ± 0.23 (0.59–1.52)	**<0**.**001**	1.01 ± 0.23 (0.55–1.47)	**<0**.**001**
RPA ratio	−0.03 ± 0.02 (−0.07–0.02)	0.230	0.04 ± 0.02 (−0.01–0.08)	0.105	0.00 ± 0.03 (−0.05–0.05)	0.985	0.01 ± 0.02 (−0.03–0.06)	0.609	0.04 ± 0.03 (−0.01–0.09)	0.147	0.01 ± 0.02 (−0.04–0.06)	0.657
SCV flow	0.14 ± 0.08 (−0.01–0.29)	0.071	0.15 ± 0.08 (−0.01–0.31)	0.064	0.11 ± 0.09 (−0.08–0.29)	0.246	0.29 ± 0.08 (0.13–0.45)	**<0**.**001**	0.26 ± 0.09 (0.08–0.44)	**0**.**004**	0.40 ± 0.09 (0.22–0.57)	**<0**.**001**
ICV flow	0.11 ± 0.10 (−0.09–0.32)	0.285	0.25 ± 0.11 (0.03–0.47)	**0**.**024**	0.08 ± 0.13 (−0.18–0.33)	0.552	0.36 ± 0.11 (0.15–0.58)	**0**.**001**	0.33 ± 0.12 (0.09–0.57)	**0**.**008**	0.44 ± 0.12 (0.20–0.68)	**<0**.**001**
Caval veins flow	0.27 ± 0.15 (−0.02–0.57)	0.072	0.39 ± 0.16 (0.08–0.71)	**0**.**015**	0.18 ± 0.18 (−0.18–0.55)	0.314	0.66 ± 0.16 (0.35–0.97)	**<0**.**001**	0.58 ± 0.18 (0.23–0.92)	**0**.**001**	0.85 ± 0.18 (0.50–1.19)	**<0**.**001**
SCV ratio	0.02 ± 0.02 (−0.02–0.06)	0.355	0.02 ± 0.02 (−0.03–0.06)	0.510	0.02 ± 0.03 (−0.04–0.07)	0.552	0.04 ± 0.02 (−0.01–0.08)	0.123	0.03 ± 0.03 (−0.02–0.08)	0.227	0.05 ± 0.03 (0.00–0.10)	**0**.**045**
Collateral flow	−0.02 ± 0.13 (−0.27–0.22)	0.849	−0.24 ± 0.13 (−0.50–0.02)	0.073	0.13 ± 0.15 (−0.17–0.42)	0.402	−0.26 ± 0.13 (−0.52–0.01)	**0**.**044**	−0.11 ± 0.15 (−0.40–0.18)	0.439	−0.14 ± 0.14 (−0.42–0.15)	0.340

CI, confidence interval; ICV, inferior caval vein; LPA, left pulmonary artery; Pulm. arteries: pulmonary arteries; RPA, right pulmonary artery; SCV, superior caval vein; T1, T2, T3, and T4, time of 1st, 2nd, 3rd, and 4th CMR.

*P*-values statistically significant are reported in bold.

A significant increase in indexed collateral flow was found comparing T1 vs. T3 (mean difference −0.26 ± 0.13, *P* = 0.04). This increase was not confirmed in T4, which showed a decrease in indexed collateral flow compared with T3, although statistically not significant (mean difference 0.13 ± 0.15, *P* = 0.40). Overall, there was no change in collateral flow (T1 vs. T4, *P* = 0.34).

The LMM regression of indexed flows and ratios at four temporal classes was repeated in the paediatric subgroup. These results were comparable with those obtained by the entire cohort of patients (see Supplementary data online, *Table S1*).

The LMM regression of indexed flows and ratios at four temporal classes was repeated in a selected subgroup of 44 patients with T1 within 10 years after Fontan completion. In general, these results were comparable with those obtained by the entire cohort of patients (see Supplementary data online, *Table S2*).

Indexed volumes and EF did not differ across the temporal classes, whilst a significant decrease in HR was found comparing T1 vs. T4 (mean difference 6.96 ± 2.51, *P* = 0.006) (*Table 5*).

**Table 5:** Linear mixed model regression for ejection fraction, heart rate, indexed end-diastolic and end-systolic volumes, and indexed stroke volume

	Mean difference between temporal classes											
	T1 vs. T2 (CI 95%)	*P*-value	T2 vs. T3 (CI 95%)	*P*-value	T3 vs. T4 (CI 95%)	*P*-value	T1 vs. T3 (CI 95%)	*P*-value	T2 vs. T4 (CI 95%)	*P*-value	T1 vs. T4 (CI 95%)	*P*-value
EF (%)	−1.54 ± 1.77 (−5.02–1.94)	0.385	−0.45 ± 1.91 (−4.21–3.32)	0.815	−1.85 ± 2.23 (−6.25–2.54)	0.498	−1.99 ± 1.91 (−5.75–1.78)	0.299	−2.30 ± 2.11 (−6.46–1.86)	0.277	−3.84 ± 2.11 (−8.00–0.32)	0.070
Heart rate (bpm)	2.08 ± 2.13 (−2.12–6.27)	0.330	1.27 ± 2.29 (−3.23–5.78)	0.578	3.61 ± 2.64 (−1.59–8.82)	0.173	3.35 ± 2.27 (−1.12–7.81)	0.141	4.89 ± 2.52 (−0.09–9.86)	0.054	6.96 ± 2.51 (2.03–11.90)	**0**.**006**
Index EDV (mL/m^2^)	3.85 ± 5.44 (−6.87–14.56)	0.480	−0.57 ± 5.88 (−12.15–11.00)	0.922	6.00 ± 6.87 (−7.52–19.52)	0.383	3.27 ± 5.88 (−8.30–14.85)	0.578	5.43 ± 6.50 (−7.37–18.22)	0.404	9.27 ± 6.50 (−3.52–22.07)	0.155
Index ESV (mL/m^2^)	2.93 ± 4.19 (−5.32–11.18)	0.485	0.04 ± 4.52 (−8.87–8.95)	0.993	5.25 ± 5.29 (−5.17–15.66)	0.322	2.97 ± 4.52 (−5.94–11.88)	0.512	5.29 ± 5.00 (−4.57–15.14)	0.292	8.22 ± 5.00 (−1.64–18.07)	0.102
Index SV (mL/m^2^)	−0.28 ± 2.20 (−4.61–4.06)	0.900	−0.80 ± 2.38 (−5.48–3.88)	0.737	1.10 ± 2.78 (−4.37–6.58)	0.691	−1.08 ± 2.38 (−5.76–3.61)	0.651	0.30 ± 2.63 (−4.88–5.48)	0.908	0.03 ± 2.63 (−5.15–5.21)	0.992

bpm, beats per minute; CI, confidence interval; EDV, end-diastolic volume; EF, ejection fraction; ESV, end-systolic volume; SV, stroke volume; T1, T2, T3, and T4, time of 1st, 2nd, 3rd, and 4th CMR.

*P*-values statistically significant are reported in bold.

The LMM regression of indexed volumes, EF, and HR at four temporal classes was repeated in the paediatric subgroup. As far as volumes and EF concern, these results were comparable with those obtained by the entire cohort of patients. A different result was found in the analysis of HR: in the paediatric population, HR did not differ across the temporal classes (see Supplementary data online, *Table S3*).

### Kaplan–Meier analysis

**Figure 2:**
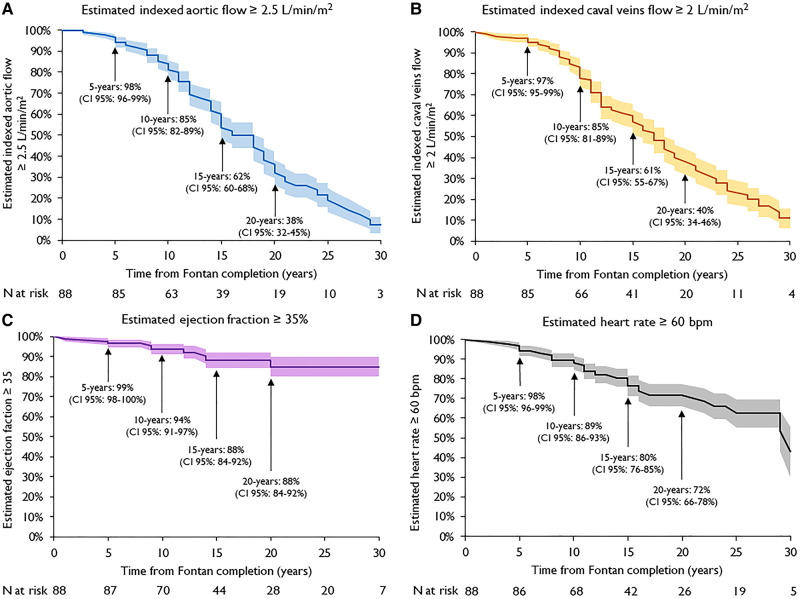
Kaplan–Meier curves of estimated indexed aortic flow ≥ 2.5 L/min/m^2^ (*A*); estimated indexed caval veins flow ≥ 2 L/min/m^2^ (*B*); estimated EF ≥ 35% (*C*); estimated HR ≥ 60 bpm (*D*). Shaded areas represent CI 95%. Time zero corresponds to date of Fontan completion.

*Figure 2A* shows the Kaplan–Meier curve for estimated indexed aortic flow ≥ 2.5 L/min/m^2^ after Fontan completion: 98% at 5 years, 85% at 10 years, 62% at 15 years, and 38% at 20 years. This degression continued up to 30 years. *Figure 2B* shows the Kaplan–Meier curve for estimated indexed caval veins flow ≥ 2 L/min/m^2^ after Fontan completion: 97% at 5 years, 85% at 10 years, 61% at 15 years, and 40% at 20 years. Also, this degression continues up to 30 years.

Estimated EF ≥ 35% at 15 years and at 20 years was 88% (*Figure 2C*), and no further changes in EF were observed even at 30-year follow-up. Estimated HR ≥ 60 bpm was 80% at 15 years and 72% at 20 years (*Figure 2D*). Similarly, to the curves of indexed aortic and caval veins flow, this degression continued at later follow-up.

**Figure 3:**
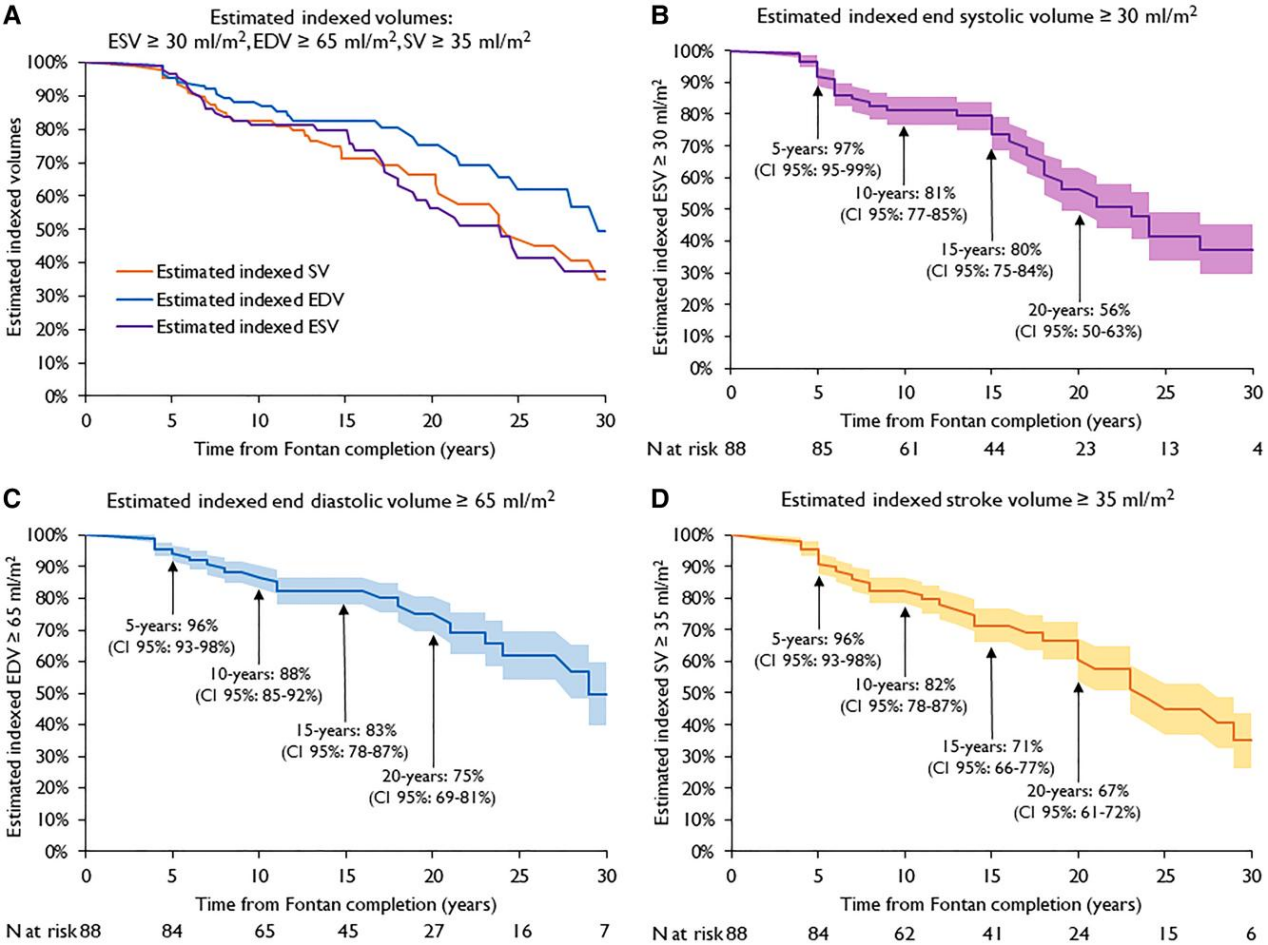
(*A*) Kaplan–Meier curves of all estimated indexed volumes: ESV ≥ 30 mL/m^2^, EDV ≥ 65 mL/m^2^, and SV ≥ 35 mL/m^2^. Kaplan–Meier curves of estimated indexed ESV ≥ 30 mL/m^2^ (*B*), EDV ≥ 65 mL/m^2^ (*C*), and SV ≥ 35 mL/m^2^ (*D*). Shaded areas represent CI 95%. Time zero corresponds to date of Fontan completion.

All Kaplan–Meier curves for the estimated indexed volumes are shown in *Figure 3A*: at 20 years, estimated indexed ESV ≥ 30 mL/m^2^ was 56% (*Figure 3B*), estimated indexed EDV ≥ 65 mL/m^2^ was 75% (*Figure 3C*), and estimated indexed SV ≥ 35 mL/m^2^ was 67% (*Figure 3D*).

At overall follow-up, 57/88 patients had an indexed aortic flow < 2.5 L/min/m^2^: 7/57 patients (12%) had an EF < 35%.

## Discussion

In this prospective, single-centre study, we analysed serial CMR in Fontan patients, and to our knowledge, this is the largest cohort of Fontan patients with serial CMR, repeated at intervals of 2–3 years across a period of 10 years. CMR is worldwide routinely performed to assess ventricular volumes, Fontan pathway patency, and flows and to evaluate atrioventricular valve regurgitation. Hence, as mentioned in the European Society of Cardiology guidelines for the management of adult congenital heart disease patients,^8^ CMR is one of the main non-invasive diagnostic tools in the workup of Fontan patients. However, these guidelines did not recommend performing serial CMR at routine practice at follow-up, which results in loss of granularity and difficulty in comparing follow-up results of Fontan cohorts amongst centres.

The significant and large decrease of indexed aortic flow and all flows of the cavopulmonary circuit with preserved ventricular EF over time, reported in this study across temporal classes, sheds light on the mechanisms of Fontan attrition. This functional decline of the Fontan circuit is the result of chronic increased systemic venous congestion, preload deprivation, and progressive increase of pulmonary vascular resistance.^9–14^ This decrease in flow continues in quasi linear fashion over the temporal observations. Therefore, in order to monitor the evolution of the univentricular circulation, we recommend serial CMR at regular intervals during follow-up.

Cardiac output reflects the basal status of the circulation, irrespective whether of the circulation is biventricular or univentricular. The indexed aortic flow measured by CMR is a similar measure as cardiac index and has therefore a particular relevance. Cardiac output is the result of the product of SV and HR, and therefore, for a thorough understanding of the degression of aortic flow, we discuss these variables separately and in more detail.

### Heart rate

A significant decrease in mean HR was registered between T1 and T4 and the Kaplan–Meier curves of indexed aortic flow and HR almost fully overlap in the first 10 years after Fontan completion (*Figure 2D*). This decrease in mean HR across the temporal classes affects the adult population, because no difference in HR was found in the paediatric subgroup. This decrease in HR differs from that of healthy adult population of comparable age. In fact, average resting HR in healthy individuals increases from 20 years old until 50–60 years old after which a downward trend begins.^15^ Furthermore, the average resting HR only falls under the threshold of 60 bpm in the oldest decades (70–80 years old).^16^ The decrease in HR could contribute to the decline of the cardiac index; however, the influence of HR on cardiac index in Fontan patients differs from the healthy population. In fact, several studies showed that in Fontan patients, increments in HR by atrial pacing resulted in decreased SV and cardiac index.^15,17^ Apparently, a decrease of diastolic filling surpassed the benefit of the increased HR. The so-called chronotropic incompetence of Fontan patients could be then re-interpreted in the light of not only the counterproductivity of further increases in HR if the cardiac index decreased but may be an attempt to compensate for diastolic dysfunction with resulting impaired filling of the systemic ventricle.^14^

### Systolic ventricular function and volumes

The systole of the sole ventricle represents the engine power of the Fontan circulation and, together with the ventricular volumes, represents a major determinant of the cardiac index. As reported before, mean EF was preserved in all temporal classes.^18^ The Kaplan–Meier curves of indexed aortic flow and EF of our study diverge remarkably. Moreover, only 12% of the patients with an indexed aortic flow < 2.5 L/min/m^2^ had an EF < 35% at overall follow-up. Therefore, the contribution of ventricular systolic dysfunction in the drop of cardiac output seems to be limited. However, diastolic dysfunction with preserved EF has been described in Fontan patients and is associated with heart failure as a result of low cardiac output and elevated end-diastolic pressure.^19^

The impaired preload and the consequent inability to increase the SV have been reported to be the main factor that determines the cardiac output, especially at exercise.^17^ In our study, no significant changes in indexed EDV, ESV, and SV were observed across the temporal classes, despite a trend of decrease either in indexed EDV or in indexed ESV.

An age-related degression in indexed EDV, ESV, and SV has been observed also in healthy individuals. However, ventricular volumes decrease slightly in healthy individuals, especially in the young adult age (up to 50 years old).^20,21^

The degression of ventricular volumes found in our Fontan patients is faster than in healthy population. In our view, it could be seen as a form of ‘premature aging’ after Fontan completion. In fact, the completion of the Fontan circulation seems to move instantaneously the range of ventricular volumes forwards towards older decades of age. This instantaneous decrease in preload volumes limits the SV through the Frank–Starling effect, whilst the potential volume of that same ventricle will then never again have a chance to be recruited for a normal cardiac output of which it is capable.

Indexed caval veins flow and pulmonary arteries flow significantly decreased across temporal classes, and the Kaplan–Meier curve of indexed caval veins flow retraces almost identically the drop of the indexed aortic flow. These results strongly suggest that pulmonary vascular resistance could negatively affect ventricular preload and cardiac output.^14^

The significant decrease in the mean SCV flow ratio across temporal classes confirmed that ICV flow represents the most important source of systemic venous return in Fontan circulation.^22^ In fact, lower ICV flow has been associated with suboptimal Fontan haemodynamics.^23^ The larger contribution of RPA to the overall pulmonary flow registered by RPA flow ratio remained constant over time and did not differ from healthy population.^6^

The role of collateral flow as a determinant of the cardiac output remains controversial, and our study does not allow us to draw strong conclusions that could clarify its role.

### Limitations

The main limitation of this study is the wide range of temporal intervals between the date of Fontan completion and the first CMR, but the effect seems to be very limited. In patients whose indexed aortic flow was <2.5 L/min/m^2^ at T1, it was not possible to determine the exact time in which it occurred. Theoretically, the indexed aortic flow of these patients could have been <2.5 L/min/m^2^ even before or at time of Fontan completion. The study cohort consists of a heterogeneous patient population as far as anatomy and surgical type of Fontan completion. This variety in baseline characteristics could have influenced some parameters of the study. However, this heterogeneity could enhance the validity of the physiological picture described in the manuscript, which could be ‘applied’ to the whole Fontan population.

## Conclusion

The degression of indexed aortic flow registered in our study reflects the attrition of the univentricular circulation that already begins within 10 years after Fontan completion. Several determinants could have contributed to the decline of the aortic flow, of which increasing pulmonary vascular resistance and chronotropic decline seem to be associated, whilst the contribution of ventricular systolic dysfunction is limited.

Based on our study, we recommend serial CMR with blood flow measurements as routine practice in the Fontan population to achieve a comprehensive and complete assessment of the univentricular physiology.

In addition, in case of worsening of the clinical picture, we recommend including CMR in the diagnostic workup. Further studies are needed to analyse the potential correlation between the measured flows and the clinical outcome of the patients.

## Supplementary Material

qyad039_Supplementary_Data

## Data Availability

The data collected in this study are saved in our Institutional Research Database and can be accessed by the co-authors of the manuscript. The data underlying this article will be shared on reasonable request to the corresponding author.

## Lead author biography

Sara Camilla Arrigoni is a congenital cardio-thoracic surgeon: born and grown in Italy; she graduated in Medicine in Milan and then moved to the Netherlands. Since 2011, she works in the Congenital Cardiac Center of the University Medical Center Groningen, the Dutch-leading medical centre for paediatric lung transplantation and multi-organ transplantation. She is pursuing a PhD investigating outcome and physiology of univentricular circulation. She is a member of the European Association for Cardio-Thoracic Surgery and European Congenital Heart Surgeons Association. Her interests include research by single ventricle patients, multi-organ transplantation, and minimally invasive surgery for congenital heart disease.
