# Supplementary material for: Cardiac output drop reflects circulatory attrition after Fontan completion: serial cardiac magnetic resonance study
Source: Eur Heart J Imaging Methods Pract. 2023 Nov 27;1(2):qyad039. doi: 10.1093/ehjimp/qyad039 (PMC11195729; doi:10.1093/ehjimp/qyad039)
Supplement: qyad039_Supplementary_Data [file qyad039_Supplementary_Data.zip › Tabel S2 mixed model Flows and Ratios within 10 years.docx]

|  | Mean difference indexed flows (L/min/m^2^) and flow ratios at temporal classes | | | | | | | | | | | |
| --- | --- | --- | --- | --- | --- | --- | --- | --- | --- | --- | --- | --- |
|  | T1 vs T2  (CI 95%) | P-value | T2 vs T3  (CI 95%) | P-value | T3 vs T4  (CI 95%) | P-value | T1 vs T3  (CI 95%) | P-value | T2 vs T4  (CI 95%) | P-value | T1 vs T4  (CI 95%) | P-value |
| **Aortic flow** | 0.39 ± 0.21  (-0.04-0.81) | 0.074 | 0.08 ± 0.24  (-0.40-0.55) | 0.746 | 0.53 ± 0.30  (-0.06-1.12) | 0.077 | 0.46 ± 0.24  (-0.01-0.94) | 0.053 | 0.61 ± 0.28  (0.05-1.16) | **0.032** | 1.00 ± 0.28  (0.45-1.55) | **<0.001** |
| **LPA flow** | 0.05 ± 0.10  (-0.14-0.24) | 0.595 | 0.20 ± 0.11  -0.01-0.41) | 0.063 | 0.14 ± 0.13  (-0.12-0.41) | 0.288 | 0.25 ± 0.11  (0.04-0.47) | **0.022** | 0.34 ± 0.12  (0.10-0.59) | **0.006** | 0.40 ± 0.13  (0.15-0.65) | **0.002** |
| **RPA flow** | -0.06 ± 0.16  (-0.39-0.26) | 0.710 | 0.55 ± 0.18  (0.20-0.91) | **0.002** | 0.01 ± 0.23  (-0.44-0.45) | 0.982 | 0.49 ± 0.18  (0.14-0.84) | **0.007** | 0.56 ± 0.21  (0.13-0.98) | **0.010** | 0.50 ± 0.21  (0.07-0.92) | **0.022** |
| **Pulm. Arteries**  **flow** | -0.17 ± 0.32  (-0.80-0.47) | 0.609 | 1.07 ± 0.36  (0.37-1.78) | **0.003** | 0.18 ± 0.44  (-0.70-1.06) | 0.682 | 0.91 ± 0.36  (0.20-1.61) | **0.012** | 1.25 ± 0.42  (0.43-2.08) | **0.003** | 1.09 ± 0.42  (0.26-1.92) | **0.010** |
| **RPA ratio** | -0.04 ± 0.03  (-0.09-0.01) | 0.133 | 0.07 ± 0.03  (0.02-0.13) | **0.013** | -0.03 ± 0.04  (-0.10-0.04) | 0.337 | 0.03 ± 0.03  (-0.02-0.09) | 0.252 | 0.04 ± 0.03  (-0.03-0.10) | 0.263 | 0.00 ± 0.03  (-0.07-0.06) | 0.965 |
| **SCV flow** | 0.24 ± 0.10  (0.03-0.44) | **0.024** | 0.08 ± 0.11  (-0.14-0.30) | 0.477 | 0.30 ± 0.14  (0.02-0.58) | **0.039** | 0.32 ± 0.11  (0.10-0.54) | **0.005** | 0.38 ± 0.13  (0.11-0.64) | **0.006** | 0.61 ± 0.13  (0.35-0.88) | **<0.001** |
| **ICV flow** | 0.10 ± 0.16  (-0.21-0.41) | 0.511 | 0.40 ± 0.17  (0.07-0.74) | **0.019** | 0.08 ± 0.21  (-0.34-0.50) | 0.712 | 0.51 ± 0.17  (0.17-0.84) | **0.003** | 0.48 ± 0.20  (0.08-0.89) | **0.019** | 0.59 ± 0.20  (0.19-0.99) | **0.004** |
| **Caval Veins flow** | 0.37 ± 0.22  (-0.08-0.81) | 0.105 | 0.45 ± 0.25  (-0.03-0.94) | 0.067 | 0.38 ± 0.31  (-0.23-0.98) | 0.219 | 0.82 ± 0.24  (0.34-1.30) | **<0.001** | 0.83 ± 0.29  (0.25-1.41) | **0.005** | 1.20 ± 0.29  (0.62-1.77) | **<0.001** |
| **SCV ratio** | 0.05 ± 0.03  (-0.01-0.10) | 0.080 | -0.02 ± 0.03  (-0.08-0.04) | 0.590 | 0.05 ± 0.04  (-0.02-0.13) | 0.169 | 0.03 ± 0.03  (-0.03-0.09) | 0.277 | 0.04 ± 0.04  (-0.04-0.11) | 0.323 | 0.08 ± 0.04  (0.01-0.15) | **0.019** |
| **Collateral flow** | 0.04 ± 0.18  (-0.31-0.40) | 0.805 | -0.38 ± 0.20  (-0.77-0.02) | 0.060 | 0.20 ± 0.25  (-0.29-0.68) | 0.427 | -0.33 ± 0.19  (-0.72-0.05) | 0.088 | -0.18 ± 0.24  (-0.65-0.28) | 0.440 | -0.14 ± 0.23  (-0.60-0.32) | 0.553 |

**Table S2. Linear mix model regression for indexed flows and ratios: 44 patients with the 1^st^ CMR within 10 years after Fontan completion**

LPA: Left Pulmonary Artery; RPA: Right Pulmonary Artery; Pulm. Arteries: Pulmonary Arteries; SCV: Superior Caval Vein; ICV: Inferior Caval Vein; vs: versus.

T1, T2, T3, T4: time of 1^st^, 2^nd^, 3^rd^, 4^th^ CMR.

CI: Confidence Interval.

P-values statistically significant are reported in bold.
