# Supplementary material for: Cardiac output drop reflects circulatory attrition after Fontan completion: serial cardiac magnetic resonance study
Source: Eur Heart J Imaging Methods Pract. 2023 Nov 27;1(2):qyad039. doi: 10.1093/ehjimp/qyad039 (PMC11195729; doi:10.1093/ehjimp/qyad039)
Supplement: qyad039_Supplementary_Data [file qyad039_Supplementary_Data.zip › Tabel S3 mixed model EF, HR and volumes - pediatric.docx]

**Table S3. Linear mix model regression for ejection fraction, heart rate, indexed end diastolic and end systolic volume, indexed stroke volume: paediatric population**

|  | Mean difference between temporal classes | | | | | | | | | | | |
| --- | --- | --- | --- | --- | --- | --- | --- | --- | --- | --- | --- | --- |
|  | T1 vs T2  (CI 95%) | P-value | T2 vs T3  (CI 95%) | P-value | T3 vs T4  (CI 95%) | P-value | T1 vs T3  (CI 95%) | P-value | T2 vs T4  (CI 95%) | P-value | T1 vs T4  (CI 95%) | P-value |
| **EF (%)** | -0.54 ± 3.01  (-6.53-5.46) | 0.859 | 1.77 ± 3.47  (-5.13-8.67) | 0.611 | -1.86 ± 4.65  (-11.11-7.39) | 0.691 | 1.23 ± 3.47  ( -5.67-8.13) | 0.723 | -0.09 ± 4.32  ( -8.69-8.51) | 0.984 | -0.62 ± 4.32  ( -9.22-7.98) | 0.886 |
| **Heart rate**  **(bpm)** | 2.75 ± 3.70  (-4.63-10.12) | 0.460 | 1.80 ± 4.25  (-6.66-10.27) | 0.673 | 3.24 ± 5.66  (-8.03-14.51) | 0.569 | 4.55 ± 4.22  (-3.86-12.96) | 0.284 | 5.04 ± 5.28  (-5.49-15.56) | 0.343 | 7.79 ± 5.26  (-2.69-18.26) | 0.143 |
| **Index EDV**  **(ml/m^2^)** | 9.90 ± 12.44  (-14.86-34.66) | 0.429 | -0.97 ± 14.31  (-29.46-27.52) | 0.946 | 0.91 ± 19.18  (-37.29-39.10) | 0.962 | 8.93 ± 14.31  (-19.56-37.41) | 0.535 | -0.06 ± 17.83  (-35.57-35.40) | 0.997 | 9.83 ± 17.83  (-25.67-45.34) | 0.583 |
| **Index ESV**  **(ml/m^2^)** | 6.97 ± 9.80  (-12.53-26.47) | 0.479 | -4.17 ± 11.27  (-26.61-18.26) | 0.712 | 1.33 ± 15.11  (-28.75-31.41) | 0.930 | 2.80 ± 11.27  (-19.64-25.23) | 0.805 | -2.84 ± 14.04  (-30.80-25.12) | 0.840 | 4.13 ± 14.04  (-23.83-32.09) | 0.769 |
| **Index SV**  **(ml/m^2^)** | 1.28 ± 4.13  (-6.94-9.49) | 0.758 | -0.49 ± 4.75  (-8.96-9.94) | 0.918 | -0.89 ± 6.37  (-13.56-11.79) | 0.890 | 1.77 ± 4.75  (-7.69-11.22) | 0.711 | -0.40 ± 5.92  (-12.18-11.38) | 0.947 | 0.88 ± 5.92  (-10.90-12.66) | 0.882 |

EF: Ejection Fraction; EDV: End Diastolic Volume; ESV: End Systolic Volume; SV: Stroke Volume; bpm: beats per minute; vs: versus.

T1, T2, T3, T4: time of 1^st^, 2^nd^, 3^rd^, 4^th^ CMR.

CI: Confidence Interval
