# Supplementary material for: Cardiac output drop reflects circulatory attrition after Fontan completion: serial cardiac magnetic resonance study
Source: Eur Heart J Imaging Methods Pract. 2023 Nov 27;1(2):qyad039. doi: 10.1093/ehjimp/qyad039 (PMC11195729; doi:10.1093/ehjimp/qyad039)
Supplement: qyad039_Supplementary_Data [file qyad039_Supplementary_Data.zip › Tabel S1 mixed model Flows and Ratios - pediatric.docx]

|  | Mean difference indexed flows (L/min/m^2^) and flow ratios between temporal classes | | | | | | | | | | | | |
| --- | --- | --- | --- | --- | --- | --- | --- | --- | --- | --- | --- | --- | --- |
|  | T1 vs T2  (CI 95%) | P-value | T2 vs T3  (CI 95%) | P-value | T3 vs T4  (CI 95%) | P-value | T1 vs T3  (CI 95%) | P-value | T2 vs T4  (CI 95%) | P-value | T1 vs T4  (CI 95%) | P-value |  |
| **Aortic flow** | 0.65 ± 0.27  (0.11-1.19) | **0.020** | 0.06 ± 0.31  (-0.57-0.68) | 0.853 | 0.72 ± 0.42  (-0.11-1.55) | 0.087 | 0.71 ± 0.31  (0.09-1.33) | **0.026** | 0.78 ± 0.39  (0.01-1.56) | **0.048** | 0.43 ± 0.39  (0.66-2.20) | **<0.001** |  |
| **LPA flow** | 0.07 ± 0.12  (-0.16-0.30) | 0.563 | 0.23 ± 0.13  (-0.04 -0.49) | 0.096 | 0.16 ± 0.18  (-0.19-0.51) | 0.370 | 0.29 ± 0.13  (0.03-0.56) | **0.032** | 0.38 ± 0.16  (0.06-0.71) | **0.021** | 0.45 ± 0.16  (0.13-0.78) | **0.007** |  |
| **RPA flow** | 0.11 ± 0.17  (-0.23-0.45) | 0.515 | 0.62 ± 0.19  (-0.23-1.00) | **0.002** | 0.03 ± 0.26  (-0.50-0.55) | 0.919 | 0.73 ± 0.19  (0.35-1.10) | **<0.001** | 0.64 ± 0.25  (0.15-1.14) | **0.012** | 0.75 ± 0.25  (0.26-1.25) | **0.003** |  |
| **Pulm. Arteries**  **flow** | -0.08 ± 0.28  (-0.65-0.48) | 0.765 | 1.09 ± 0.33  (0.44-1.74) | **0.001** | 0.22 ± 0.44  (-0.66-1.11) | 0.620 | 1.00 ± 0.32  (0.36-1.65) | **0.003** | 1.31 ± 0.41  (0.48-2.14) | **0.002** | 1.23 ± 0.41  (0.40-2.05) | **0.004** |  |
| **RPA ratio** | -0.02 ± 0.03  (-0.09-0.04) | 0.447 | 0.08 ± 0.04  (-0.00-0.15) | **0.037** | -0.04 ± 0.05  (-0.14-0.06) | 0.418 | 0.05 ± 0.04  (-0.02-0.13) | 0.144 | 0.04 ± 0.05  (-0.06-0.13) | 0.427 | 0.01 ± 0.05  (-0.08-0.11) | 0.780 |  |
| **SCV flow** | 0.29 ± 0.14  (0.02-0.56) | 0.035 | 0.02 ± 0.15  (-0.28-0.33) | 0.880 | 0.46 ± 0.21  (-0.04-0.88) | **0.034** | 0.32 ± 0.15  (0.01-0.62) | **0.043** | 0.48 ± 0.20  (0.08-0.88) | **0.019** | 0.77 ± 0.20  (0.38-1.17) | **<0.001** |  |
| **ICV flow** | 0.30 ± 0.17  (-0.04-0.65) | 0.080 | 0.36 ± 0.19  (-0.02-0.75) | 0.065 | 0.24 ± 0.26  (-0.29-0.76) | 0.376 | 0.67 ± 0.19  (0.29-1.05) | **<0.001** | 0.60 ± 0.25  (0.10-1.10) | **0.019** | 0.90 ± 0.25  (0.41-1.40) | **<0.001** |  |
| **Caval Veins flow** | 0.61 ± 0.26  (0.10-1.12) | **0.019** | 0.37 ± 0.29  (-0.21-0.95) | 0.208 | 0.70 ± 0.40  (-0.09-1.48) | 0.082 | 0.98 ± 0.29  (0.41-1.55) | **<0.001** | 1.07 ± 0.37  (0.32-1.81) | **0.006** | 1.68 ± 0.37  (0.94-2.42) | **<0.001** |  |
| **SCV ratio** | 0.03 ± 0.03  (-0.04-0.10) | 0.371 | -0.02 ± 0.04  (-0.10-0.06) | 0.592 | 0.07 ± 0.05  (-0.04-0.18) | 0.197 | 0.01 ± 0.04  (-0.07-0.09) | 0.795 | 0.05 ± 0.05  (-0.05-0.15) | 0.341 | 0.08 ± 0.05  (-0.02-0.18) | 0.116 |  |
| **Collateral flow** | 0.06 ± 0.25  (-0.43-0.55) | 0.803 | -0.33 ± 0.28  (-0.88-0.23) | 0.245 | 0.13 ± 0.38  (-0.62-0.88) | 0.738 | -0.26 ± 0.27  (-0.81-0.28) | 0.334 | -0.20 ± 0.36  (-0.92-0.52) | 0.579 | -0.14 ± 0.35  (-0.84-0.57) | 0.697 |  |

**Table S1. Linear mix model regression for indexed flows and flow ratios: paediatric population**

LPA: Left Pulmonary Artery; RPA: Right Pulmonary Artery; Pulm. Arteries: Pulmonary Arteries; SCV: Superior Caval Vein; ICV: Inferior Caval Vein; vs: versus.

T1, T2, T3, T4: time of 1^st^, 2^nd^, 3^rd^, 4^th^ CMR.

CI: Confidence Interval.

P-values statistically significant are reported in bold.
